# A Research Agenda for Malaria Eradication: Drugs

**DOI:** 10.1371/journal.pmed.1000402

**Published:** 2011-01-25

**Authors:** The malERA Consultative Group on Drugs

## Abstract

Antimalarial drugs will be essential tools at all stages of malaria elimination along the path towards eradication, including the early control or “attack” phase to drive down transmission and the later stages of maintaining interruption of transmission, preventing reintroduction of malaria, and eliminating the last residual foci of infection. Drugs will continue to be used to treat acute malaria illness and prevent complications in vulnerable groups, but better drugs are needed for elimination-specific indications such as mass treatment, curing asymptomatic infections, curing relapsing liver stages, and preventing transmission. The ideal malaria eradication drug is a coformulated drug combination suitable for mass administration that can be administered in a single encounter at infrequent intervals and that results in radical cure of all life cycle stages of all five malaria species infecting humans. Short of this optimal goal, highly desirable drugs might have limitations such as targeting only one or two parasite species, the priorities being *Plasmodium falciparum* and *Plasmodium vivax*. The malaria research agenda for eradication should include research aimed at developing such drugs and research to develop situation-specific strategies for using both current and future drugs to interrupt malaria transmission.

## Abstract 2

The Malaria Eradication Research Agenda (malERA) Consultative Group on Drugs present a research and development agenda to ensure that appropriate drugs are available for use in malaria eradication.

## Introduction

Antimalarial drugs are used to treat malaria illness, to prevent both infection and disease caused by *Plasmodia*, to eliminate dormant malaria parasites from the liver, and to prevent malaria transmission. In the context of malaria elimination or eradication, drugs have been used for both treatment and prevention in situations where intensive surveillance has been used to identify cases, and in mass drug administration (MDA) programmes without regard for the presence of infection.

The malERA Drugs Consultative Group brought together malaria biologists, drug developers, clinical investigators, and control officials, and consulted outside experts on drug development and disease eradication to identify and prioritize a preliminary set of knowledge gaps and research questions that need to be addressed to use drugs effectively along with other tools to eliminate and ultimately eradicate malaria. The consultative process was predicated on several key assumptions, and included a review of the role of drugs in past and recent elimination campaigns.

Several current research questions were identified that should be high priorities whether or not malaria eradication moves forward. However, the main work of the group was to draft a research and development agenda that focuses on those new research questions and knowledge gaps that arise specifically in response to the call for malaria eradication and that would not otherwise be at the top of the malaria research agenda. Thus, new and better drugs for intermittent preventive treatment (IPT) of malaria in pregnancy and molecular markers that can be used as surveillance tools for monitoring artemisinin-resistant malaria are both critically important research priorities, but are not specific to the malaria eradication agenda, and are not discussed in this paper.

In this paper, “eradication” refers to the interruption of transmission and fall in disease incidence to zero worldwide, “elimination” refers to interruption of transmission and a fall in disease incidence to zero in a defined geographical area, and “control” refers to reduction of disease incidence and burden to the point where it is no longer a public health priority.

## Starting Assumptions

The thinking of the malERA Drugs Consultative Group was based on the assumption that malaria eradication is not possible with existing tools, which include artemisinin-based combination treatments (ACTs), long-lasting insecticide-treated nets, and insecticide spraying. It is true that with this set of tools, dramatic reductions in malaria have been achieved recently in many countries, including some in Africa [1]. Malaria has even been completely eliminated from some areas with low levels of transmission and relatively sound health care infrastructure by the World Health Organization (WHO) Global Malaria Eradication Program and by more recent elimination efforts [2]. However, it is the view of the malERA Drugs Consultative Group that complete global malaria eradication will not be accomplished within most of our lifetimes, and that new tools, including new antimalarial drugs developed specifically for elimination indications, are essential to move towards and ultimately achieve this ambitious but eminently worthy goal. Our thinking was also predicated on the assumption that these new tools will need to be used in combinations with each other.

The reasons for these first two starting assumptions include the critical fact that eradication entails the complete elimination of any latent or persistent parasite reservoir in the human population. The complex life cycles of the five malaria species infecting humans present different challenges. Malaria parasites can persist for years without causing symptoms, both in the liver (in the case of *P. vivax* and *Plasmodium ovale*), and in the blood (*Plasmodium malariae*), and low-level infections that cannot be detected by standard diagnostic methods can nevertheless propagate transmission. Eradication of targeted malaria species is therefore likely to require drugs that can accomplish complete “eradication” of every malaria parasite from the bodies of infected humans, including those who are carrying very low levels of parasites that cause no symptoms but that might be a source of transmission. Moreover, we anticipate that eradication tools are likely to become increasingly compromised by the emergence and spread of drug-resistant [3] and “vaccine-resistant” parasites [4] and of insecticide-resistant mosquitoes [5], and we recognize that tools and approaches that were successful in settings with reasonably intact health care systems, functioning governments, and accessible populations, will be inadequate for the elimination of malaria in the hardest-to-reach and most unstable corners of the malaria-endemic world.

Moreover, although insecticides are appropriately credited for much of the success of the first global eradication campaign carried out in the mid-20th century, careful review of malaria control and elimination efforts shows that treatment and prevention with drugs have also been essential components of all successful malaria elimination schemes. Similarly, although smallpox has been eradicated and polio nearly so primarily through the use of vaccines, for reasons elucidated elsewhere [6], including the partial and temporary nature of naturally acquired protective immunity to malaria, and the need to eliminate latent infections that persist in the face of natural immunity, it is very unlikely that malaria could be eradicated with even a highly efficacious vaccine without concomitant use of drugs and antivector methods. The notion that a single silver bullet in the form of one brilliant technological advance could spell the end of the single biggest killer of human beings for thousands of years is appealing, but borders on magical thinking. We fervently hope to be proven wrong on this point, and strongly encourage young scientists to pursue brilliant technological advances and silver bullets, but believe that investment in a variety of complementary tools is needed.

Another of our starting assumptions was that although the current scheme for malaria elimination described in the Global Malaria Action Plan [7] calls for the elimination stage to begin when control efforts result in a reduction in malaria incidence to <1 case/1,000 population at risk, malERA should consider research questions related to the possible role of new and old drugs at all stages of malaria control and elimination. In particular, the role of drugs in aggressive efforts to drive down high transmission rates during the control phase of eradication—formerly and perhaps more inspirationally called the “attack” phase—should be considered. For example, drugs might be used in mass screening and treatment or MDA campaigns, or as ongoing IPT intended to reduce both morbidity and transmission.

Finally, we assumed that incipient elimination and eradication efforts will likely focus initially chiefly on *P. falciparum* in Africa, but that *P. vivax* will be a major focus outside of Africa, where it is the most common form of malaria. *P. vivax* causes more morbidity, severe disease, and death than is often appreciated [8]. It also presents special challenges because of the relapsing liver-stage parasites (hypnozoites) that are refractory to treatment with most antimalarial drugs, and it will increase in prominence as rates of falciparum malaria decrease. We therefore assumed that research and development will proceed in parallel to develop drugs that can be used to eliminate *P. falciparum* and *P. vivax*, ideally drugs that target both species, although better species-specific drugs are also likely to make a great contribution. Eventually, the other human malarias, *P. malariae*, *P. ovale*, and *Plasmodium knowlesi*, may have to be considered as specific targets for global eradication as their impact is modified by control or elimination of the other species, although it is hoped that these minority species will be eliminated in a collateral fashion by tools aimed at falciparum and vivax malaria.

## What's New about the Approach to Drugs in the Context of Elimination?

In the first malaria eradication campaign, antimalarial drugs were considered for their role in eliminating infection in people and thus reducing the infectious reservoir. Subsequently, there was a reorientation towards thinking about controlling malaria as a disease rather than as an infection, with more emphasis on preventing clinical complications and death [9]. There was less concern about curing infection in settings where rapid re-infection was guaranteed. To prepare for a long-term approach to elimination, it is necessary to revive the earlier paradigm and again think about malaria drugs and other interventions in terms of their impact on malaria infection and transmission in addition to their use in the prevention and treatment of malaria disease. “Elimination thinking” also underlies the concept of adding anti-gametocytocidal drugs to the treatment of malaria in areas such as Cambodia [10] where resistance to ACT drugs is being observed [11],[12].

### Current Drug Indications

We identified several high priority research areas that need to be addressed urgently regardless of whether the world mobilizes for a renewed effort to eliminate malaria. The first such area is optimization of the use of ACTs and other currently available antimalarial drugs to maximize their useful lifespan. Approaches to achieve this optimization include the rational design of drug combinations with well-matched pharmacokinetic and pharmacodynamic profiles, operational research to increase uptake of coformulated ACTs while minimizing the use of artemisinin monotherapy and suboptimal dosing, and the evaluation of strategies to reduce relative pressure for emergence and dissemination of resistance [13],[14].

The second high priority area is continued research and development to make new drugs available to replace current drugs (in particular, artemisinins) as resistance emerges. Specific priorities include first-line drugs for treating uncomplicated falciparum and vivax malaria, drugs to treat severe malaria, drugs for IPT of infants, pregnant women and children, drugs for travel chemoprophylaxis, and anti-relapse drugs to cure the liver stages of *P. vivax*.

Research is also needed to elucidate the pharmacokinetics and pharmacodynamics and optimal dosing of drugs used to treat and prevent malaria, especially in understudied vulnerable groups including pregnant women, young children and infants, as is operational research and research into improved diagnostics, and into monitoring to optimize drug deployment strategies and facilitate control efforts using currently available antimalarial drugs.

Although malERA's charge was to identify new research questions and knowledge gaps that arise in response to the call for malaria eradication, several of these research areas—for example, maintaining the development pipeline of first-line drugs to treat uncomplicated falciparum malaria—will have to be addressed for eradication to succeed. But, while it is extremely important that this pipeline continues to flow whether or not global malaria eradication is being attempted, the malERA Drugs Consultative Group did not focus on defining the optimal characteristics of drugs for treating this or other clinical malaria syndromes. Instead, we focused on drugs that would be needed specifically for the purposes of eradication, noting, for example, the need for widespread use (possibly in whole asymptomatic populations) of drugs with better safety profiles than would be required for treatment of individuals with potentially life-threatening clinical malaria.

### New Drug Indications in the Context of Eradication

Table 1 lists the current indications for antimalarial drugs, and considers the relevance of these for the specific goal of malaria eradication. For example, suppressive prophylaxis, which prevents malaria disease but that does not prevent and may even augment transmission, is not a relevant indication for malaria eradication.

**Table 1:** Indications for antimalarial drugs in the present control era and their relevance in the eradication era.

Indications for Antimalarial Drugs in the Control Era	Relevant to Malaria Eradication?
Prophylaxis	
Causal prophylaxis^a^	Yes, completely blocks infection and thus transmission
Suppressive prophylaxis^b^	No, does not prevent, and may augment, transmission
IPT of pregnant women, infants, or children	Maybe, but only if transmission-blocking drugs are used in a high proportion of the infected reservoir, essentially amounting to intermittent MDA
Treatment of disease	
Uncomplicated malaria	
*P. falciparum* and *P. malariae*	Maybe, treatment indications for specific clinical syndromes are not directly relevant to the goal of eradication unless treatment drugs have transmission-blocking efficacy; widespread use of treatment drugs with antiliver stage and gametocytocidal activity would contribute to transmission reduction.
*P. vivax* and *P. ovale*	As above
Severe malaria	As above
Antihypnozoite (liver-stage radical cure)	Yes, high priority
Transmission blocking	Yes, high priority

a Causal prophylaxis targets pre-erythrocytic liver stages and, if effective, prevents any parasites from reaching the blood state or being transmitted to mosquitoes.

b Suppressive prophylaxis is repeated subcurative dosing that suppresses blood-stage infection and prevents malaria illness but does not eradicate malaria infection or prevent transmission

It is reasonable to assume that drugs that target *P. falciparum* will generally be effective against *P. malariae*, and that those targeting *P. vivax* will be efficacious against *P. ovale* and *P. knowlesi*. This assumption is based on limited experience with current drugs, and should be tested by routinely including patients infected with these minority species in drug trials. No single trial would include enough cases of the minority species to provide a meaningful measure of efficacy, but pooling data from many trials using a global database such as the Worldwide Antimalarial Resistance Network (WWARN; www.wwarn.org) [15] would, over time, permit estimation of efficacy of commonly used drugs against these species. Importantly, the recent identification of the monkey malaria *P. knowlesi* as a widespread and potentially life-threatening human pathogen [16] suggests that vigilance for transfer of other nonhuman primate malarias to humans and the determination of which drugs are effective against these emerging diseases may be necessary in the late stages of elimination.

## Lessons Learned from Past Malaria Elimination Programmes and Efforts to Eradicate Other Diseases

As a matter of priority, experienced malariologists need to dedicate substantial time and effort to detailed analytical reviews of published and unpublished information on past elimination efforts. Here we briefly summarize a few of the insights gained from malERA reviews of some of the available material, including a dissection of the Global Malaria Eradication Program [17], and a broad overview of lessons learned from past malaria elimination efforts published by the Malaria Elimination Group [18]. In particular, we note the need for a much more comprehensive review of the use of drugs in past elimination efforts, which includes careful analysis of factors leading to success or failure (see Table 2).

**Table 2:** Past use of drugs in malaria elimination.

How Drugs Were Used in Elimination	General Assessment
Curative therapy for cases detected through surveillance	Essential component of all successful control and elimination programmes
Intensive, multidrug, multidose MDA used in conjunction with aggressive antivector interventions (nets, spraying, larvicides)	Contributed to several successful elimination programmes
Less intensive MDA as a complement to less aggressive or subsequent antivector interventions	Limited, transient, or no success at elimination
MDA as the main elimination measure	Successful only in a few cases of isolated, stable populations
Medicated salt	Mixed success and major drawbacks of danger of rapid selection for resistance and safety issues

### Importance of Single-Encounter Therapy

For smallpox, the only infectious disease that has been eradicated, a single-dose vaccine was available that could emulate the lifelong protective immunity that results from natural infection. Similarly, the drugs that are presently being used in large-scale infectious disease control and elimination programmes such as those for onchocerciasis and trachoma can be administered in a single encounter once or twice a year. Discussions with leaders of these campaigns highlighted the notion that single-encounter interventions are an essential requirement for successful elimination campaigns. Notably, however, the antimalarial drug regimens that were used with varying success to eliminate malaria from Italy [19], the former Soviet Union [20], and various islands such as the Vanuatu island of Aneityum [21], have involved complex regimens of multiple administrations of at least two drugs usually repeated at frequent intervals for prolonged periods of time. We concluded therefore that while multiple dosing regimens of multiple drugs have been used successfully to eliminate malaria from areas with relatively good health systems and stable populations, malaria eradication will require drugs that, ideally, can be administered in a single encounter at infrequent intervals (see Box 1).

**Box 1. Single Encounter Radical Cure and Prophylaxis (SERCaP)**

Currently, the goal of antimalarial drug therapy is to reduce disease and death by targeting blood-stage parasites, with an emphasis on falciparum malaria in young children in Africa. This goal is accomplished by prompt diagnosis and treatment of fever with effective drugs such as ACTs. For eradication to be effective, drug therapy must eliminate the human reservoir of infection, an objective that is best achieved by Single Encounter Radical Cure and Prophylaxis (SERCaP). Achieving the objective in a single patient encounter is important for effectiveness. Radical cure is defined as eliminating all parasites in the patient; eradication of the disease on a population basis can only be achieved by “eradication” of the parasites in all individuals. For *P. falciparum*, this entails the elimination of all persistent asexual blood-stage forms, and the long-lived mature-stage V *P. falciparum* gametocytes that are responsible for transmission. For *P. vivax* malaria, radical cure includes elimination of all persistent asexual blood-stage forms, and the long-lived hypnozoites in the liver. Finally prophylaxis highlights the need to prevent reinfection of each individual treated for some defined period after treatment. This time should be at least for 1 month, to outlast the typical development period of *Plasmodium* parasites in Anopheline mosquitoes.

### Mass Drug Administration

MDA refers to the use of drugs to treat whole populations for malaria, irrespective of, and without knowledge of, who is infected [22]. Although this approach is not currently recommended, antimalarial drugs have been used in MDA campaigns since at least 1900, when subsidized and free quinine was distributed by the Italian government for both suppressive prophylaxis and curative treatment [19]. Suppressive prophylaxis reduces the risk of acute malaria illness by controlling the level of infection without ridding the body of parasites; curative treatment resolves an acute malaria illness episode by eliminating all asexual blood-stage malaria parasites and may or may not result in a fully sterilizing cure; both approaches may either prevent, augment, or have no effect on transmission to mosquitoes. The Italian MDA campaign resulted in large decreases in malaria cases and mortality but not interruption of transmission [19]. Malaria was only finally eliminated in Italy when DDT spraying was aggressively deployed after World War II in combination with systematic diagnosis and quinine treatment and mass quinine prophylaxis.

In the former Soviet Union, mass chemoprophylaxis with blood schizonticides (drugs that kill the blood-stage malaria parasites that cause disease but that do not usually affect either liver-stage parasites or the sexual stage gametocytes that transmit malaria to the mosquito) was administered each year at the peak of the malaria season during the attack phase of elimination, then phased out during the “consolidation” phase as the last remaining foci of transmission were extinguished [20]. As malaria transmission risk coalesced into localized “islands” of risk, the entire local population was given both a blood schizonticide and an 8-aminoquinoline 2–3 weeks before the start of the malaria season. 8-aminoquinolines are active against gametocytes as well as against *P. vivax* and *P. ovale* relapsing liver forms; examples of 8-aminoquinolines include plasmocide and quinocide (now superseded drugs that were used in the USSR), the widely used and licensed primaquine, and tafenoquine, which is still investigational.

Other examples of MDA campaigns include the Garki Project in Nigeria, where simultaneous spraying and MDA significantly but only transiently reduced malaria parasite prevalence rates [23]. Similarly, mass chemoprophylaxis of a million soldiers used in conjunction with insecticide-treated nets and spraying resulted in the near-elimination of vivax malaria where it had reemerged 20 years after the Korean demilitarized zone had been declared malaria free [24]. Most recently, mass administration of artemisinin, piperaquine, and primaquine in Cambodia resulted in dramatic reductions in the prevalence of *P. falciparum*, *P. vivax*, and *P. malariae*, including a 10-fold reduction in the prevalence of falciparum gametocytes, but not the complete interruption of transmission [10]. Often, these schemes were implemented with no clear idea of what the MDA programme was trying to achieve, and in many cases political or economic factors were major drivers [22]. However, the main factors that are common to successful MDA schemes include a careful preparatory phase, social mobilization, improvement of the health care infrastructure and the inclusion of malaria control in comprehensive health care, and the concomitant use of antivector measures.

Another common success factor is that MDA (like other malaria elimination efforts) is more likely to work where malaria risk is circumscribed, such as on sea islands or in “islands” of malaria risk surrounded by areas with no malaria. Sustained interruption of falciparum and vivax malaria transmission was achieved in 1996 on the Vanuatu island of Aneityum with an intensive MDA regimen consisting of weekly chloroquine and primaquine for 9 weeks, sulfadoxine-pyrimethamine at weeks 1, 5, and 9, and concomitant use of insecticide-treated nets and larvicides [21]. A less intensive MDA regimen using three drugs at two-monthly intervals followed by DDT spraying at the end of the campaign had no measurable impact on overall malaria prevalence on the island of Zanzibar [25], highlighting the need to deploy multiple interventions aggressively and simultaneously to interrupt transmission. Where there are large areas of contiguous malaria risk, as in much of sub-Saharan Africa, the effectiveness of MDA has been transient at best. However, high transmission intensity does not necessarily preclude successful use of MDA; rather, high transmission often signifies contiguity with surrounding areas of malaria risk, with inevitable back-flow of infections unless MDA and other interventions are applied widely and simultaneously across the entire area of contiguous risk through transnational cooperation, another factor that is common to successful MDA programmes.

Finally, although MDA in the form of adding antimalarial drugs to salt used for cooking and flavoring food had some success in reducing malaria prevalence in large-scale pilot programmes in Asia, Africa, and South America [26], the inability to control dosage and the resulting rapid selection for drug-resistant parasites make this an unjustifiable approach [22].

### Long-Acting Formulations

Another creative approach from the past that may hold promise for the future is the use of long-acting formulations. “Repository” formulations of malaria drugs to provide prolonged protection were extensively researched in the early 1960s [27], and oil-based depot injections of cycloguanil pamoate provided more than 1 year of protection against experimental challenge with *P.* falciparum sporozoites [28]. These injections were evaluated in at least 15,000 people, but never deployed as a tool for elimination because of the attendant pain and local abscesses.

## Key Knowledge Gaps and Research Priorities

On the basis of this initial review of past and present malaria control and elimination efforts, the malERA Drugs Consultative Group concluded that antimalarial drugs will be essential components for elimination of malaria from endemic countries and eventually for worldwide eradication. In the next step of our discussions, we identified the key knowledge gaps about the role of drugs in malaria eradication and research priorities for developing and using drugs in malaria elimination and eradication programmes. We organized these knowledge gaps into three areas: (1) the optimization of the use of currently available drugs for elimination and eradication; (2) the development of new drugs for elimination and eradication; and (3) the development of drug treatment and prevention strategies for elimination and eradication. The rest of this paper considers these areas, which together make up the draft research and development agenda that we propose in Box 2. Finally, we also briefly touch on cross-cutting issues that require coordination with the other malERA groups.

**Box 2. A Draft Research and Development Agenda for Drugs for Malaria Eradication**

**Knowledge gaps and research priorities for optimizing current drugs**

- Pharmacology studies to optimize dosing regimens of 8-aminoquinolines for gametocytocidal and anti-relapse efficacy and safety
- Rapid and robust point-of-care glucose-6-phosphate dehydrogenase (G6PD) test to improve safety of 8-aminoquinoline use
- Tests that can detect resistance to artemisinins and ACT partner drugs
- Determine gametocytocidal and anti-relapse activity of current drugs and those in the pipeline

**Knowledge gaps and research priorities for developing new drugs for malaria eradication**

***Desired products***

- Drugs that prevent transmission by killing or preventing development of gametocytes, or blocking sporozoite development in the mosquito
- Drugs that cure liver stages of vivax (and ovale) malaria
- Ideally, drugs that can be administered in a single encounter at infrequent intervals, and that result in radical cure of all parasite stages (Single Encounter Radical Cure and Prophylaxis, see Box 1)
- Sustained or pulsed release formulations
- Exceptionally safe schizonticidal drugs for curing asymptomatic falciparum infection

***Fundamental research questions aimed towards developing desired drugs***

- Fundamental studies of liver and sexual stage biology (in both host and mosquito)
- Mechanisms of resistance and pharmacological strategies to deter resistance
- In vitro culture of *P. vivax* to understand parasite biology

***Tools and capacities***

- Increased capacity for clinical pharmacology research including pharmacokinetics/pharmacodynamics studies in populations targeted for malaria elimination
- Increased capacity for human challenge studies for early go/no go decisions on drug candidates
- Assays to measure transmission-blocking activity
- Assays to measure activity against liver stages
- In vitro culture of *P. vivax* and other non-falciparum species for drug screening
- Genomic and proteomic approaches to identify transmission-blocking and liver-stage activity

**Knowledge gaps and research priorities for drug treatment and prevention strategies for eradication**

- Field studies to evaluate new drugs and approaches in a variety of epidemiological settings
- Robust and highly sensitive malaria diagnostics for malaria infection and especially for carriage of infectious gametocytes
- Measures to monitor and improve adherence and safety
- How must drug treatment and prevention strategies change as elimination proceeds?
- Strategies to deter resistance

## Optimization of the Use of Currently Available Drugs for Elimination

The time from lead identification of a new compound to a licensed drug is measured in decades. Thus, the optimization of existing tools for control and elimination must occur in parallel with development of new tools for elimination and eradication. As discussed earlier, one of the assumptions underlying the malERA process is that global eradication of malaria cannot be accomplished with existing tools, but that malaria is being eliminated from areas with relatively low transmission and relatively good health systems using these tools. Consequently, in parallel with the development of new drugs and other eradication tools, research is needed to optimize drugs that can be used now to reduce malaria transmission. The first section of Box 2 highlights priority knowledge gaps and research questions related to currently available antimalarial drugs. Most of these topics should already be research priorities irrespective of malaria eradication. They are highlighted here because they are essential for eradication but relatively neglected. The most important knowledge gaps relate to the use of 8-aminoquinolines and ACTs. 8-aminoquinolines are the only drugs available today that can kill dormant liver stages and gametocytes. Primaquine, the only currently licensed 8-aminoquinoline, is routinely used to prevent relapses of *P. vivax* and *P. ovale* and has played a prominent role in several successful elimination campaigns; the long-acting 8-aminoquinoline tafenoquine is not yet licensed. ACTs, which are presently the first-line treatment for both uncomplicated and severe falciparum malaria in most of the world, are threatened by the recent emergence of artemisinin resistance in Southeast Asia [12],[29].

Research to optimize the successful use of these drugs to eliminate malaria from the approximately 30 countries now actively pursuing this goal represents “low-hanging fruit” that is likely to yield high gains at relatively low cost over the next 5–10 years. While recognizing that global eradication will require substantial investment in new tools, the large gains that can be made and consolidated by making the most of the tools now in hand should not be underestimated.

The paucity of information about pharmacokinetics, pharmacodynamics, and rational dosing of drugs represents a critical knowledge gap that needs to be addressed in order to use current drugs in conjunction with other tools to reduce malaria transmission, as well as to provide rationally designed treatment strategies. The other top priority is the development of robust and sensitive field diagnostics to guide drug interventions and to detect carriage of gametocytes that are infectious to mosquitoes. This type of research is also a priority for vaccine development [30].

## Development of New Drugs for Elimination and Eradication

The second section of Box 2 summarizes key knowledge gaps and research priorities for the development of new drugs specifically for elimination and eradication indications. Below, we discuss some of these issues in more detail. Importantly, because antimalarial drugs have not previously been licensed for indications other than individual treatment, early and close consultation with regulatory authorities will be needed for any drugs used for elimination and eradication.

### Targeting Liver and Sexual Stages and Greater Emphasis on Safety

In 1957 Wallace Peters wrote, “Development of an 8-aminoquinoline in depot form to give a safe and adequate blood level should be attempted as this would be an invaluable weapon against malaria if properly applied” [31]. More than 50 years later, the dream of a safe, long-acting drug that eliminates malaria infection by killing liver stages and that blocks transmission by killing gametocytes remains both unfulfilled and a top priority. As mentioned earlier, the only known antimalarial drugs that kill dormant liver stages and gametocytes are the 8-aminoquinolines primaquine and tafenoquine. Both of these drugs have a serious flaw for a drug that would be used to eliminate infection and block transmission in people who are not themselves acutely sick with malaria—they cause hemolysis (destruction of red blood cells leading to anemia) in individuals with glucose-6-phosphate dehydrogenase (G6PD) deficiency, a red cell polymorphism that is common in tropical populations because it is associated with some degree of protection against malaria illness [32]. Any drug used for malaria elimination in people who are not sick must have a low risk-to-benefit ratio akin to the low risk-to-benefit ratios of routine immunizations.

During its brainstorming sessions, the malERA drugs group developed draft target product profiles (TPPs) for new drugs that could be used for radical cure (including elimination of both liver stages and gametocytes) of *P. falciparum* and *P. vivax*. These TPPs (see Tables S1–S3) represent the ideal targets and a starting point for discussion with drug developers. Drugs that fall short of these ideals will still be of value for eradication, and adjudicating between the ideal and the acceptable will be a dynamic and continuous process. For example, the ideal drug would target all malaria species, but it would not be prudent to reject promising candidates that target only *P. falciparum* or *P. vivax*. Indeed, depending on leads and progress, it is likely to be necessary to pursue at least partly separate research agendas for these two key species.

### Ideal Pharmacokinetic/Pharmacodynamic Characteristics

An ideal eradication drug would have a short half life with a sustained (depot-like) release followed by rapid elimination, and would be deployed in combination with other drugs with matching pharmacokinetic and pharmacodynamic profiles both to deter resistance and to improve efficacy [13]. Such characteristics would allow for rapid onset of action to exert quick killing, long duration of action to permit administration in a single encounter at infrequent intervals, and rapid clearance to avoid a long period of sublethal drug levels conducive to selection for resistant parasites. An intermediate goal may be to develop a safe product for delivery at a single encounter of a curative dose of a drug that also offers 4 or more weeks of post-treatment causal prophylactic efficacy.

Antimalarial drugs with half lives in the range of weeks are already available but do not offer the ideal pharmacokinetic/pharmacodynamic profile of sustained or intermittent pulsed killing levels followed by rapid drop-off to deter resistance. Previous research on polymers for pulsed release of malaria vaccines showed initial promise but was abandoned by WHO. Nanotechnology may offer another approach for developing drugs with the ideal pharmacokinetic/pharmacodynamic profile. Nanoparticle delivery of drugs and vaccines is in the early stages of development, and one challenge for this form of delivery is the limitation on the dose of drug that can be delivered. Highly potent drugs that require a low dose would therefore be most attractive for this delivery method. Subcutaneous implants such as those used to deliver birth control drugs also warrant consideration.

The malERA drugs group felt strongly that concerns of impracticality and expense should not deter research into the ideal pharmacokinetic/pharmacodynamic profile. Seemingly risky approaches can and should be entertained in the quest for solutions and evaluated for their potential. Speculation about the ultimate cost of an intervention should not be the sole basis for its rejection from further consideration.

In some circumstances, these ideal pharmacokinetic/pharmacodynamic characteristics may be less critical, such as when drugs are administered in settings or at times when there is very low or no transmission. A specific example of this is the use of mass treatment to eliminate the infectious reservoir at the nadir of malaria transmission in settings with sharply seasonal malaria transmission. Because there is a very low probability of parasites encountering subtherapeutic drug levels in this setting, there may be little disadvantage to drug combinations with mismatched pharmacokinetic/pharmacodynamic profiles. This example highlights the importance of considering setting-specific epidemiology and indications when thinking about desired characteristics of drugs for elimination and supports the idea of tailoring TPPs to specific indications on the basis of the parasites, human populations, epidemiological settings, and stages of elimination and eradication that are to be targeted.

### Drug Resistance

It has been suggested that concerns about drug resistance and strategies to deter it—for example, the obligatory use of combinations of drugs with different mechanisms—may not be a priority in the context of malaria eradication, because resistance is unlikely to emerge and spread when transmission is very low in the late stages of elimination. However, evidence suggests that drug resistance can spread rapidly and become fixed in populations in settings of low malaria transmission [33], and history amply demonstrates the folly of counting on the efficacy of drugs to endure in the face of widespread use [3]. Even at “the last mile,” when eliminating the last few cases of malaria from an area, the risk of exporting malaria to, or reintroducing it from, other malarious areas will remain. This risk will only disappear during the final stages of global eradication when remaining foci are very few and far between. For these reasons, it is important that the development process for drugs and drug combinations for elimination and eradication indications should attempt to build in strategies for preventing and deterring resistance. These strategies include combining drugs with different mechanisms of action [34] or even drugs with opposing resistance mechanisms, and coformulation of drugs.

### Clinical Research

The current capacity for conducting both laboratory and clinical malaria pharmacokinetics/pharmacodynamics studies is very limited. Consequently, most antimalarial drugs are used in risk groups and populations for whom there is little to no information on optimal dosing for efficacy and safety. Careful and rigorous clinical pharmacology studies will be needed for new drugs and drug combinations for eradication, and robust methods instituted for postlicensure marketing surveillance for side effects. This research will require expanded capacity for drug level measurements, pharmacokinetics analysis and clinical pharmacology studies, and surveillance. As malaria incidence falls at established malaria research sites, it is already becoming increasingly difficult to meet sample size requirements for drug efficacy trials. It may, therefore, become necessary to establish mobile clinical trial networks or novel clinical trials designs (for example, field trials in malaria-exposed populations that measure gametocyte prevalence and infectivity) to assess the efficacy of drugs for blocking transmission and preventing relapse and/or to rely more on the use of experimental malaria challenge studies [35] to evaluate drugs (and vaccines) for eradication.

## Drug Treatment and Prevention Strategies for Eradication

Although much can be learned from careful review of the role played by drugs in past elimination programmes, creatively designed prospective field research and pilot projects and operational research to assess interventions as they are implemented in different settings will be essential for the success of malaria eradication (see the final section of Box 2). That is, research is needed to understand when, where, and how to use drugs to eliminate and eradicate malaria. For example, current guidelines do not recommend MDA, and evidence from field studies of the efficacy of specific interventions in specific populations and epidemiological settings is needed to support a change in this recommendation. Thus, the effectiveness of mass screening and treatment of only infected individuals needs to be compared with treating all individuals irrespective of whether they are infected, as is done in MDA. Similarly, the effectiveness of “focal screening and treatment,” (a variation on mass screening and treatment that uses molecular diagnostics to identify the individuals to be given curative treatment) that is now being used in an attempt to contain emerging artemisinin-resistant falciparum malaria in western Cambodia [36] needs to be properly evaluated.

Furthermore, research is needed into the different drug treatment and prevention strategies that will be needed for different epidemiological settings at different stages of the elimination process, and in settings with different levels of health care infrastructure. Drug treatment and prophylaxis schemes that are feasible and effective in stable rural populations with year-round malaria transmission may be completely ineffective if implemented in a setting with highly seasonal malaria, or impossible in mobile populations or in areas of civil unrest. Moreover, as transmission rates decline, so will levels of protective immunity, resulting in fewer cases of infection spread across a wider range of age groups. As reduced transmission is sustained for years, asymptomatic carriage will become increasingly uncommon, making MDA less attractive [37].

Research is also needed into robust and sensitive screening tests to guide drug treatment and prophylaxis both for asexual parasites and for infectious gametocytes and to evaluate the efficacy of drugs (and vaccines) that are intended to block transmission. The current gold standard, light microscopy, is insufficiently sensitive to detect low levels of gametocytes that are nevertheless capable of being transmitted, and current investigational assays that offer improved sensitivity are not robust enough for field surveillance in most settings, nor are they validated as predictive of infectivity.

## Cross-Cutting Issues

Several important knowledge gaps and research priorities for drug strategies cut across one or more of the technical areas covered by malERA. For example, for any malaria research enterprise to succeed, it is essential to engage scientists in endemic countries to identify, prioritize, and refine research questions and to design the most appropriate approaches. This process is particularly important for research involving human-based interventions such as drugs, because issues like population acceptance, adherence, and impact of epidemiological differences on drug efficacy and safety require knowledge of local cultural, political, ecological, and epidemiological factors. Other examples of cross-cutting research issues include research into health systems and how they deliver malaria interventions, operational research, malaria modeling and research into monitoring and surveillance, vaccines, and vector control. These cross-cutting issues are addressed in the other malERA papers in this series [30],[37]–[40].

## Concluding Remarks

The potential list of research priorities for developing and using drugs to eradicate malaria is as long as the list of research interests of the individuals who participated in the consultative process. To be useful in setting a research agenda for eradication, however, the list must be focused and prioritized. This report focuses on research goals that will be achieved largely in the longer term, and these suggestions are passed to the rest of the malaria community in the form of a draft research and development agenda (Box 2) with a sincere request that the whole of the malaria community uses its considerable wisdom and experience to improve this agenda in the spirit of a shared hope for a malaria-free future.

## Supporting Information

Table S1 TPP for drugs used to treat and prevent infection (prophylaxis) in elimination programmes: Single Encounter Radical Cure and Prophylaxis against all parasitic species (SERCaP).
(0.05 MB DOC)

Table S2 Short of Single Encounter Radical Cure and Prophylaxis against all parasitic species (SERCaP), TPP for drugs used for radical cure of *P. falciparum* in elimination programmes.
(0.04 MB DOC)

Table S3 Short of Single Encounter Radical Cure and Prophylaxis against all parasitic species (SERCaP), TPP for drugs used for radical cure of *P. vivax* in elimination programmes.
(0.04 MB DOC)

## Abbreviations

ACT: artemisinin-based combination therapy
IPT: intermittent preventive treatment
MDA: mass drug administration
TPP: target product profile
