# Supplementary material for: A Research Agenda for Malaria Eradication: Drugs
Source: PLoS Med. 2011 Jan 25;8(1):e1000402. doi: 10.1371/journal.pmed.1000402 (PMC3026688; doi:10.1371/journal.pmed.1000402)
Supplement: Table S1 — TPP for drugs used to treat and prevent infection (prophylaxis) in elimination programmes: Single Encounter Radical Cure and Prophylaxis against all parasitic species (SERCaP). (0.05 MB DOC) [file pmed.1000402.s001.doc]

**Supplementary Tables: Target Product Profiles**

**Supplementary Table 1. Target product profile for drugs used to treat and prevent infection (prophylaxis) in elimination programs: Single Encounter Radical Cure and Prophylaxis against all parasitic species (SERCaP)**

| **Key Product Characteristics** | **SERCaP (Single Encounter Radical Cure and Prophylaxis) against all parasitic species** | | |
| --- | --- | --- | --- |
| **Indication** | **Intermittent (continuous)[[1]](#footnote-2) prophylaxis, giving four weeks of complete protection** | **Post-exposure prophylaxis** | **Radical cure of symptomatic or asymptomatic malaria** |
| **Class / mechanism of action** | Active against all stages  (causal only) | Active against all stages including established hypnozoites | Active against all stages including gametocytes and established hypnozoites |
| **Requires combination**  **with other drugs** | Yes, to ensure efficacy and deter resistance | Yes, to ensure efficacy and deter resistance | Yes, to ensure efficacy and deter resistance |
| **Formulation** | Pediatric suitable, co-formulated | Pediatric suitable, co-formulated | Pediatric suitable, co-formulated |
| **Pharmacokinetics/pharmacodynamics (PK/PD) of the combination** | Consistent with six monthly (weekly) dosing | Consistent with single (3 daily) dosing | TBD |
| **Route of administration** | Oral (intramuscular, implant, subcutaneous, transdermal) | Oral (IM, implant, SC, transdermal) | Oral, or parenteral for acutely ill, |
| **Dosing regimen** | Single dose (up to 3 daily doses if infrequent dosing) | Single dose (up to 3 daily doses if infrequent dosing) | Single dose one dose for all age groups (3 doses) |
| **Efficacy at preventing all stages** | ~100% for six months (95% for one week) | ~100% (95%) | 100% |
| **Efficacy against gametocyte and hypnozoites (transmission blocking potential)** | ~100% (95%) | ~100% (95%) | ~100% (95%) |
| **Safety** | At least as good as current prophylaxis drugs[[2]](#footnote-3) in all populations, including pregnant women and infants. Does not require pre-treatment screening for Mass Drug Administration. | At least as good as current prophylaxis drugs^2^ in all populations, including pregnant women and infants. Does not require pre-treatment screening for Mass Drug Administration. | Better than current drugs[[3]](#footnote-4) for treatment and radical cure in all populations, including pregnant women and infants. Does not require pre-treatment screening for Mass Drug Administration. |
| **Safety duration** | 2 years (3 months) | 2 years (3 months) | 2 years (3 months) |
| **Shelf life (years)** | 5 (2) | 5 (2) | 5 (2) |
| **Packaging & labeling** | TBD | TBD | TBD |
| **Susceptibility to resistance** | Active against resistant parasites | Active against resistant parasites | Active against resistant parasites |

Note: The ideal drug would be effective against all species. Drugs effective for either *P. falciparu*m or *P. vivax* alone have potential to make a large impact.

PK; pharmokinetic; PD: pharmodynamic; IM: intramuscular; SC subcutaneous; TBD: to be determined

1. Parentheses indicate acceptable, as opposed to ideal, characteristics. [↑](#footnote-ref-2)
2. Atovaquone/proguanil, doxycycline, mefloquine [↑](#footnote-ref-3)
3. ACT plus primaquine [↑](#footnote-ref-4)
