# Supplementary material for: A Research Agenda for Malaria Eradication: Drugs
Source: PLoS Med. 2011 Jan 25;8(1):e1000402. doi: 10.1371/journal.pmed.1000402 (PMC3026688; doi:10.1371/journal.pmed.1000402)
Supplement: Table S2 — Short of Single Encounter Radical Cure and Prophylaxis against all parasitic species (SERCaP), TPP for drugs used for radical cure of P. falciparum in elimination programmes. (0.04 MB DOC) [file pmed.1000402.s002.doc]

**Supplementary Table 2. Short of SERCaP, target product profile for drugs used for radical cure of *P. falciparum* in elimination programs**

| **Key Product Characteristics** | **Benchmark[[1]](#footnote-2)** | **Minimally acceptable criteria** | **Ideal criteria** |
| --- | --- | --- | --- |
| **Primary indication** | Treatment of uncomplicated malaria (ill patients) | Treatment of asymptomatic parasitemia (well people) | Radical cure of *P. falciparum*: Treatment of asymptomatic parasitemia plus stage V gametocytes |
| **Class / mechanism of action** | Active against asexual stages (10-40 hours) plus stage I-IV gametocytes | Active against all stages | Active against all stages |
| **Requires combination**  **with other drugs** | Yes, to ensure efficacy and deter resistance | Yes, to ensure efficacy and deter resistance | Yes, to ensure efficacy and deter resistance |
| **Formulation** | Pediatric suitable, co-formulated | Pediatric suitable, co-formulated | Pediatric suitable, co-formulated |
| **PK/PD of the combination** | various | TBD | TBD |
| **Route of administration** | Oral | Oral | Oral |
| **Dosing regimen** | Various: once daily x 3 doses and AL 6 x 60 | Single dose (3 days). One dosing across all age groups (4 or less) | SERCaP One dosing across all age groups (4 or less) |
| **Efficacy against asexual stages** | >95% (>90%) PCR corrected day 28  (survival analysis) | >95% (>90%) PCR corrected day 28  (survival analysis) | >95% (>90%) PCR corrected day 42  (survival analysis) |
| **Efficacy against gametocytes (transmission blocking potential)** | Stages I-IV | Stages I-V | stages I-V |
| **Safety** | Known | Better than (as good as) standard radical therapy (ACT + primaquine), in most populations, Does not require pre-screening | Better than (as good as) standard radical therapy (ACT + primaquine), in all populations, including pregnant women. Does not require pre-screening. High tolerability |
| **Shelf life (years)** | ?? | 5 (2) | 5 (2) |
| **Packaging & labeling** | Various | TBD | TBD |
| **Susceptibility to resistance** | Various | Active against resistant parasites | Active against resistant parasites |

1. Benchmark: Best product currently in use = an effective ACT [↑](#footnote-ref-2)
