# Supplementary material for: A Research Agenda for Malaria Eradication: Drugs
Source: PLoS Med. 2011 Jan 25;8(1):e1000402. doi: 10.1371/journal.pmed.1000402 (PMC3026688; doi:10.1371/journal.pmed.1000402)
Supplement: Table S3 — Short of Single Encounter Radical Cure and Prophylaxis against all parasitic species (SERCaP), TPP for drugs used for radical cure of P. vivax in elimination programmes. (0.04 MB DOC) [file pmed.1000402.s003.doc]

**Supplementary Table 3. Short of SERCaP, target product profile for drugs used for radical cure of *P. vivax* in elimination programs**

| **Key Product Characteristics** | **Benchmark[[1]](#footnote-2)** | **Minimally acceptable criteria** | **Ideal criteria** |
| --- | --- | --- | --- |
| **Primary indication** | Radical cure of *P. vivax* | Radical cure of *P. vivax* | Radical cure of *P. vivax* |
| **Class / mechanism of action** | Active against all stages | Active against all stages | Active against all stages |
| **Requires combination**  **with other drugs** | Yes, to ensure efficacy and deter resistance | Yes, to ensure efficacy and deter resistance | Yes, to ensure efficacy and deter resistance |
| **Formulation** | TBD; possible co-formulation with a *P. falciparum* combination | TBD; possible co-formulation with a *P. falciparum* combination | TBD; possible co-formulation with a *P. falciparum* combination |
| **PK/PD of the combination** | Not known | TBD | TBD |
| **Route of administration** | Oral | Oral | Oral |
| **Dosing regimen** | Single daily dose of PQ X at least 14 days | Single dose (3 days) | Single dose (SERCaP) |
| **Efficacy against asexual stages** | >95% (>90%) PCR corrected day 28  (survival analysis) | >95% (>90%) PCR corrected day 28  (survival analysis) | >95% (>90%) PCR corrected day 42  (survival analysis) |
| **Efficacy against hypnozoites** | Dose, weight, and strain dependent | Effective in all geographic areas with a total weight based dose given in 3 days | Effective in all geographic areas with a total weight based dose given in 1 dose |
| **Efficacy against gametocytes (transmission blocking potential)** | Not known for *P. vivax* (may not be important) | Not known for *P. vivax* (may not be important) - TBD | Not known for *P. vivax* (may not be important) - TBD |
| **Safety** | G6PD deficiency liabilities | Better than (as good as) standard radical therapy (ACT + primaquine), in most populations. Does not require pre-screening. | Better than (as good as) standard radical therapy (ACT + primaquine), in all populations, including pregnant women. Does not require pre-screening. High tolerability. |
| **Shelf life (years)** | Not known | 5 (2) | 5 (2) |
| **Packaging & labeling** | Various | TBD | TBD |
| **Susceptibility to resistance** | Active against resistant parasites | Active against resistant parasites | Active against resistant parasites |

1. Benchmark for *P. vivax* radical cure is combination chloroquine plus primaquine, except where chloroquine-resistant *P. vivax* necessitates use of ACT plus primaquine. [↑](#footnote-ref-2)
